# The effect of HIV educational interventions on HIV-related knowledge, condom use, and HIV incidence in sub-Saharan Africa: a systematic review and meta-analysis

**DOI:** 10.1186/s12889-018-6178-y

**Published:** 2018-11-13

**Authors:** Lena Faust, Sanni Yaya

**Affiliations:** 1 Faculty of Health Sciences, University of Ottawa, Ottawa, Canada; 2 School of International Development and Global Studies, University of Ottawa, 120 University, Ottawa, ON K1N 6N5 Canada

**Keywords:** HIV, HIV-related knowledge, Meta-analysis, Sub-Saharan Africa

## Abstract

**Background:**

As high stigmatization of HIV and relatively low knowledge of HIV transmission and prevention measures persist in Sub-Saharan Africa, the improvement of HIV-related knowledge, and the evaluation of which types of interventions are most effective in this regard, is an important aspect of further prevention efforts. In addition, it is of interest to assess whether improvements in HIV-related knowledge may actually lead to increased engagement in preventive behaviours and ultimately lower HIV transmission. This study therefore aims to systematically review and meta-analyse the evidence for the effect of HIV-related knowledge interventions on 1) the improvement of HIV-related knowledge, 2) subsequent risk reduction behaviour (condom use), 3) lower incidence of HIV infection.

**Methods:**

A literature search was conducted using the Embase and Medline databases, returning 746 after duplicate removal. Following abstract and full-text screening, 36 studies were ultimately included in the final review. Meta-analyses were conducted in R, using random-effects models, for the HIV-related knowledge, condom use, and HIV incidence outcomes, where sufficient data were available.

**Results:**

Interventions assessed in the reviewed studies varied, including computer-based interventions, mass media campaigns, and peer education interventions. The interventions were generally found to be effective at improving HIV-related knowledge in the target population, with 10 studies reporting improved knowledge of risk reduction through condom use in the intervention group (out of 11 studies reporting data for this outcome), with 6 reporting these differences as significant (*p* < 0.05). Regarding knowledge of transmission routes, studies assessing peer education interventions often reported significant improvements in the intervention group. Meta-analysis results showed significantly higher odds among the intervention groups of correct knowledge of: risk reduction through condom use (OR: 3.09, 95%CI: 1.83–5.22, *p* < 0.0001), sexual transmission of HIV (OR: 5.86, 95%CI: 2.65–12.97, *p* < 0.001) and transmission through sharps (OR: 4.35, 95%CI = 3.21–5.90, *p* < 0.001), but non-significantly lower odds of HIV infection (OR: 0.97, 95%CI: 0.66–1.41, *p* = 0.854).

**Conclusion:**

Peer-education-based interventions appear to be particularly effective in facilitating the uptake of HIV-related knowledge, particularly pertaining to transmission routes. There is some evidence that improved knowledge of HIV transmission and prevention facilitates increased subsequent engagement in preventive measures, although this requires further exploration.

**Trial registration:**

PROSPERO Number: CRD42018090600

**Electronic supplementary material:**

The online version of this article (10.1186/s12889-018-6178-y) contains supplementary material, which is available to authorized users.

## Background

Given that Sub-Saharan Africa accounts for more than 70% of worldwide HIV cases [1], investigating effective approaches to HIV prevention in the area remains urgent. As high stigmatization of the disease and relatively low knowledge of HIV transmission and prevention measures in the region persist [2], the improvement of HIV-related knowledge is an important aspect of further prevention efforts [3–5], as an understanding of one’s risk of contracting or transmitting the disease, as well as an understanding of effective preventive measures has the potential to increase engagement in such measures [6], and in turn reduce future transmission.

Thus far, efforts towards improving HIV-related knowledge in Sub-Saharan Africa have encompassed a wide variety of intervention types and methods of disseminating HIV-related information, such as peer education [7–9], game-based education [10], skill-building interventions [11, 12], and mass media campaigns [13]. Many such interventions have drawn on various theoretical frameworks, such as social cognitive theory [8, 14], the theory of planned behaviour [12], and the theory of reasoned action [15, 16]. Recognizing the significant social determinants of the disease, including in particular the roles of gender inequality and female disempowerment in the continued transmission of HIV, educational interventions focusing on HIV prevention have often also been designed with reference to social and gender-inequity-based theories such as the theory of gender and power, social norms theory, and the social constructivist theory of gender [17]. Moreover, interventions based on theories of behaviour change have also been commonly used in interventions aiming to improve HIV-related knowledge, as it has been found that HIV education interventions are associated with a greater likelihood of subsequent adoption of preventive behaviours when implemented in combination with behaviour change elements [18].

Therefore, although a number of studies in Sub-Saharan Africa have reported on a wide variety of different types of HIV-related knowledge interventions, their effectiveness in the Sub-Saharan population has not yet been systematically compared. Moreover, given that specific socio-demographic risk groups for low HIV-related knowledge have been identified in previous studies, such as a recent study investigating socio-demographic predictors of HIV-related knowledge in Nigeria [2], it is of interest to examine whether certain interventions are particularly effective among specific strata of the population. This information will aid the further design or adaptation of interventions aimed at improving HIV-related knowledge in the region, and will allow more targeted resource-allocation to the types of interventions or methods of dissemination that have been found to be most effective.

Lastly, as a number of studies have assessed HIV-related knowledge in various countries across sub-Saharan Africa and South Asia, but many of these did not examine whether higher HIV-related knowledge is actually associated with a lower likelihood of HIV infection [2, 5, 19], this review will serve as a synthesis of evidence regarding the extent to which improvements in HIV-related knowledge actually lead to increased engagement in preventive behaviours, and subsequently a lower likelihood of HIV infection.

This study will therefore systematically review and meta-analyse the evidence for the influence of HIV-related knowledge interventions on 1) the improvement of HIV-related knowledge, 2) subsequent adoption of risk reduction behaviour (condom use), and 3) incidence of HIV infection.

## Methods

### Search strategy and study registration

A literature search of the Embase and Medline databases was conducted in November 2017, using the search term outlined in detail in Additional file 1: Table S1. The conduct of this systematic review is reported according to the PRISMA reporting guidelines for systematic reviews and meta-analyses [20], and the study protocol is registered on the PROSPERO database, available here.

### Eligibility criteria

Eligibility for inclusion in the review were primary, original research studies published in French or English, reporting on the implementation of a HIV-related knowledge intervention in Sub-Saharan Africa. HIV-related knowledge interventions included any interventions that aimed at improving any aspect of HIV-related knowledge, which could include, for example, knowledge of HIV prevention or transmission, or HIV risk reduction interventions. Excluded studies were those not taking place in Sub-Saharan Africa, qualitative studies, those not administering an intervention or program aimed at improving HIV-related knowledge (e.g. cross-sectional studies on HIV-related knowledge, general sexual health interventions not specific to HIV, or assessments of knowledge of HIV status only), or those targeting HIV educational interventions at healthcare providers. Also excluded were conference abstracts, editorials, commentaries, study protocols, news articles, and secondary analyses (e.g. reviews or meta-analyses).

### Study selection

Abstracts were screened according to the aforementioned criteria, and full-texts were retrieved for eligible studies. At full-text review, in addition to the abovementioned criteria, studies were excluded if they did not report quantitative data on at least one of the following outcomes of interest: a) changes in HIV-related knowledge (see Table 1. for included knowledge questions), b) adoption of preventive measures (condom use) or c) HIV incidence. Note that regarding preventive behaviours, only reported condom use was considered an outcome of interest, whilst mere *intention* to do so was not. Studies that did not provide the following for at least one of the outcomes of interest were also excluded: a) pre- and post-intervention data, or b) control group vs. intervention group data.

**Table 1:** HIV-related knowledge questions considered in this review

Knowledge Category	Question (correct answer)
Risk Reduction	Using condoms correctly can reduce one’s risk of HIV infection (yes)
Transmission Modes	HIV can be transmitted by sexual activity (including oral, anal and genital sex) (yes)
	HIV can be transmitted through contact with an infected individual’s blood (yes)
	HIV can be transmitted through sharing sharp objects such as needles with infected individuals (yes)

### Data collection and risk of Bias assessment

The Covidence systematic review management platform (Covidence systematic review software, Veritas Health Innovation, Melbourne, Australia) was used for study de-duplication and screening. Data extraction was carried out in Microsoft Excel (Version 14.5.5). Risk of bias for RCTs and non-RCTs was assessed using the 9-item Cochrane Risk of Bias Tool [21], and risk of bias in uncontrolled (e.g. one-arm pre-post studies) was assessed based on the Quality Assessment Tool for Before-After (Pre-Post) Studies With No Control [22]. These tools assess the methodological quality of studies based on criteria such as the random allocation of participants to control or intervention groups, blinding of participants and outcome assessors, cross-contamination of study groups, attrition bias, and bias in outcome reporting.

### Data analysis

For the primary outcome of interest, HIV-related knowledge, data relating to two domains of HIV-related knowledge were extracted: 1) knowledge of HIV risk reduction through condom use 2) knowledge of modes of transmission (through blood and sexual contact). The specific questions for which data were extracted are shown in Table 1. These knowledge areas are used in the analysis due to their relevance to the secondary outcome of condom use. Data were extracted either as continuous data, in the form of mean knowledge scores, or as categorical data, in the form of proportions of respondents providing correct answers. If studies reported proportions for incorrect answers, the data was transformed to reflect the proportion with correct answers, in order to maintain continuity of outcome reporting across studies. Where it was unclear whether the study was reporting negative or affirmative answers for a question, the outcome in question was not extracted for this study. Similarly, if multiple knowledge points were combined in a single question (e.g. HIV can be contracted through sexual intercourse and through the sharing of needles), data for this question was not extracted.

Secondary outcomes considered in this review were condom use and HIV incidence. Condom use was measured as defined by the study (e.g. proportion always using condoms, proportion using condoms at last sex, or over a specified time period).

Meta-analyses were conducted in cases where more than 2 studies reported sufficient data on an outcome of interest. Categorical data (e.g. proportion of participants having correct knowledge) are meta-analysed using odds ratios as the effect size measure. (In the case of zero-value cells, a decimal close to zero was substituted to allow estimation of the odds ratio). Where possible, analyses were grouped by intervention type, in order to more clearly display the effect of specific interventions types, rather than only the pooled effect of any intervention. All meta-analyses were carried out in R (specifically the metafor package) [23], using a random-effects model, as the true effect size was expected to vary between studies, given the different locations and target populations of the included studies. (Note that for random effects models, inverse-variance-based weighting is employed, according to *w*_*i*_ = 1/(τ^2^ + *v*_*i*_), where τ^2^ is the between-study variance).

## Results

### Search results

**Fig. 1:**
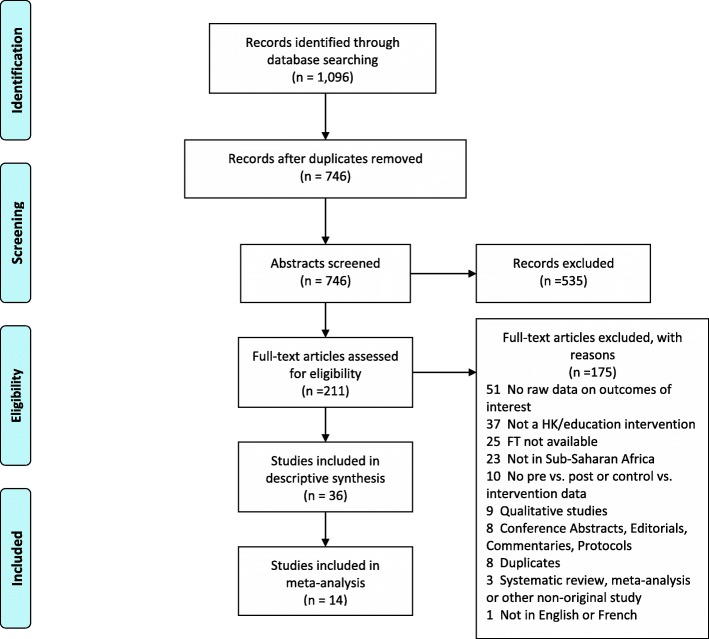
PRIMSA flowchart

The literature search (see Additional file 1 for search term) returned 1096 studies, with 746 remaining after duplicate removal. Five hundred thirty-five studies were excluded after abstract screening, and thus 211 full articles were screened. Following 175 exclusions at full-text level (mainly due to not reporting raw data on the outcomes of interest (*n* = 51), or not reporting on a HIV-related knowledge intervention (*n* = 37)) 36 studies were included in the final review. Figure 1 shows the study screening and inclusion process.

### Study characteristics

Of the 36 included studies, 12 were conducted in South Africa, 8 in Nigeria, 3 in Zimbabwe, and 3 in Kenya. Other study countries included Uganda (*n* = 2), Rwanda (*n* = 2), Zambia, Angola, Mozambique, Malawi, Madagascar, and Senegal (*n* = 1 each). Interventions in the included studies targeted a variety of populations, with the ages of study participants ranging from 10 to 70 years. Unfortunately, only 5 studies reported the baseline HIV status of their study population, with 4 reporting both HIV+ and HIV- participants, and 1 being conducted only in HIV+ individuals (31 studies did not provide data on baseline HIV status). Most studies were randomized controlled trials (RCTs) (*n* = 12), 9 were one-arm repeated measures studies (pre-post) (whilst one provided only post-intervention data), 7 were quasi-RCTs, 3 were cluster RCTs, 2 were non-randomized controlled trials, one was described as a quasi-experimental study, and one as a matched control study. Study sample sizes ranged from 60 to 11,448 participants. The oldest study was published in 1990, and the most recent in 2016. Additional file 1: Table S2 provides further details on study characteristics.

Specific types of interventions administered included peer education interventions (*n* = 8), community education interventions (*n* = 2), scenario-based interventions (*n* = 2), and integrated psychosocial or psychological interventions along with HIV education (*n* = 2). Other intervention types included audio-based interventions (*n* = 1), mass media campaigns (*n* = 1), and radio-based interventions. In addition, several studies combined multiple intervention components or approaches (*n* = 7). For example, peer education and videos were used in combination with other intervention components or mediums among the included studies (*n* = 3, and *n* = 1, respectively). The different components of the interventions implemented in each included study are summarized in Additional file 1: Table S3. Educational interventions that did not involve a particular medium, defining element or specific approach other than the communication of HIV/AIDS information (e.g. through lectures) were classified as educational / informational interventions (*n* = 5). Most (*n* = 25) interventions were implemented at the group-level, whilst 4 were targeted at individuals, 3 had both individual and group components, 1 was aimed at couples, and 3 were mass campaigns or community-level interventions. The length of group or individual intervention programs varied between one-hour one-time interventions to interventions that lasted up to 2 years with multiple sessions over this time, whilst mass campaigns tended to be longer (lasting up to 4 years).

### Intervention effects on HIV-related knowledge

#### Knowledge of risk reduction through condom use

This outcome assessed whether respondents knew that using condoms during sex reduces one’s risk of HIV infection. Results for this outcome are shown in Table 2. Eleven studies reported data on knowledge of condom use as a measure of HIV risk reduction, with 6 reporting significant increases in respondents with correct knowledge in the intervention group. These 6 studies assessed peer or community education interventions (*n* = 3) [24–26], general educational/informational intervention programs (*n* = 2) [27, 28], and video-based interventions (*n* = 1) [29], thus aligning well with the findings of other studies in this review, which report generally successful outcomes in peer-led interventions, as mentioned below [24, 25, 30].

**Table 2:** Intervention Effects on Proportions of Respondents with Correct Knowledge of Risk Reduction through Condom Use

Study (Ref, Year)	Study Country	Int Type	Sample Size (N)					*P* Value Type^a^	Proportion with Correct Answer to Risk Reduction Question (N) (unless otherwise stated)					
			Total	int bsl	cont bsl	int fu	cont fu		int bsl	cont bsl	*P* value	int fu	cont fu	*P* value
Using condoms during sexual intercourse can reduce the risk of HIV transmission (True)														
[35] 2013	Nigeria	CE	60	60		60		NA	41		NR	42		NR
[26] 1994	South Africa	CE	567	231	336	206	276	I	74	101	0.617	92	75	0.001
[36] 2013	South Africa	Comp / game	253	195		195		P	156		0.5	157		
[27] 1995	Zimbabwe	Ed	285	141	144	141	144	I	112	82	NR	113	64	< 0.001
[37] 2012	South Africa	Ed	130	130		130		NA	52		NR	72		NR
[28] 2006	Zimbabwe	Ed	869			251	618	I				123	229	< 0.001
[13] 2006	Nigeria	MM	6000	6000		NR		NA	86.2%	93.2%	NR	89.1%	95%	NR
[25] 2014	Nigeria	PE	400	200	200	195	192	P	139	146	0.0001	169	145	0.66
[24] 2007	Nigeria	PE / drama	1029	591	438	588	430	P	290	244	< 0.001	443	250	NR
[29] 2016	Mozambique	Video	915	NR	NR	462	453	I	NR	NR	NR	425	362	< 0.001
[38] 1999	Nigeria	Video / scenario	450	233	217	223	210	NR	NR	NR	NR	215	132	NR

*Bsl* baseline, *CE* Community Education, *Comp* Computer-based intervention, *Cont* Control, *Drama* drama-based intervention, *Ed* Educational / informational, *Fu* Follow-up, *MM* Mass Media, *Int* Intervention, *NR* Not reported, *PE* Peer education, *Scenario* scenario-based/ role-play based

^a^ *P* value type I = Int. vs. control, P = Pre- vs. Post-test. Where two *p* values are available, Int. vs. cont *p* values: 1st *p* value = Int, 2nd = Control. Pre-Post *p* values: 1st p value = pre-test, 2nd = post-test

#### Transmission knowledge

Overall, 11 studies assessed knowledge of sexual transmission, 5 assessed knowledge of transmission via blood, and 7 assessed knowledge of transmission through contact with infected sharps (e.g. needles). Table 3 summarizes the results of intervention effects on knowledge of transmission modes of HIV. For knowledge of transmission through sexual contact, all studies that showed significant improvement in the intervention group were studies whose intervention contained peer education elements [24, 25, 30]. Notably, for knowledge of transmission through contact with infected blood, all 5 studies that reported on this outcome reported improvement in the intervention group, but this was only stated as significant in one study, whilst the remaining studies did not report *p*-values [25].

**Table 3:** Intervention Effects on Proportions of Respondents with Correct Knowledge of Modes of Transmission of HIV

Study (Ref, Year)	Study Country	Int Type	Sample Size (N)					*P* Value Type^a^	Proportion with Correct Answer to Transmission Route Question (N) (unless otherwise stated)					
			Total	int bsl	cont bsl	int fu	cont fu		int bsl	cont bsl	*P* value	int fu	cont fu	*P* value
HIV can be transmitted through sexual contact (True)														
[35] 2013	Nigeria	CE	60	60		60		NA	50		NR	55		NR
[39] 2016	Madagascar	Ed	155	28		28		P	0.64 (0.49) ^b^		NR	0.64 (0.49)^b^		
[40] 2011	South Africa	Ed	103	58	45	58	45	NA	32	30	NR	38	23	NR
[27] 1995	Zimbabwe	Ed	285	141	144	141	144	I	125	123	NR	127	104	ns
[13] 2006	Nigeria	MM	6000	6000		NR		NA	79.5%		NR	86.3%		
[30] 2013	Nigeria	PE	160	80	80	80	80	I	70	34	< 0.001	60	34	< 0.001
[25] 2014	Nigeria	PE	400	200	200	195	192	P	184	182	< 0.001	194	178	0.82
[41] 2012	Kenya	PE	442			145	297	NA			NR	124	200	NR
[42] 2000	Senegal	PE	260	247		247		NA	235		NR	240		NR
[24] 2007	Nigeria	PE / drama	1029	591	438	588	430	P	426	307	< 0.001	582	322	NR
[38] 1999	Nigeria	Video / scenario	450	233	217	223	210	NA	192	183	NR	220	178	NR
HIV can be transmitted through contact with infected blood (True)														
[35] 2013	Nigeria	CE	60	60		60		NA	48		NR	55.98		NR
[40] 2011	South Africa	Ed	103	58	45	58	45	NA	49	33	NR	58	45	NR
[13] 2006	Nigeria	MM	6000	6000		NR		NA	24.90%		NR	29.80%		
[25] 2014	Nigeria	PE	400	200	200	195	192	P	186	176	0.02	192	173	0.71
[38] 1999	Nigeria	Video / scenario	450	233	217	223	210	NA	NR	NR	NR	210.066	166	NR
HIV can be transmitted through contact with contaminated sharps (e.g. needles) (True)														
[43] 2010	Nigeria	Aud	1205	595	560	513	461	I	524	439	< 0.0001	483	373	0.0003
[35] 2013	Nigeria	CE	60	60		60		NA	49		NR	56		NR
[40] 2011	South Africa	Ed	103	58	45	58	45	NA	49	35	NR	58	44	NR
[37] 2012	South Africa	Ed	130	130		130		NA	101		NR	104		NR
[13] 2006	Nigeria	MM	6000	6000		NR		NA	39.10%		NR	46.50%		
[25] 2014	Nigeria	PE	400	200	200	195	192	P	180	178	0.03	188	159	0.24
[38] 1999	Nigeria	Video / scenario	450	233	217	223	210	NA	NR	NR	NR	197	129	NR

*Aud* Audio-based, *Bsl* baseline, *CE* Community Education, *Comp* Computer-based, *Cont* Control, *Ed* Educational / informational, *Fu* Follow-up, *Int* Intervention, *MM* Mass Media, *NR* Not reported, *PE* Peer education, *Scenario* scenario-based /role-play based

^a^ *P* value type I = Int. vs. control, P = Pre- vs. Post-test. Where two *p* values are available, Int. vs. cont *p* values: 1st *p* value = Int, 2nd = Control. Pre-Post *p* values: 1st *p* value = pre-test, 2nd = post-test

^b^ Mean (SD)

### Intervention effects on condom use

Condom use was defined in several ways (e.g. number of unprotected sex acts) and over various time points, depending on the study. Results regarding mean condom use are shown in Additional file 1: Table S4, and categorical data for condom use (e.g. proportion of respondents reporting consistent condom use) are shown in Additional file 1: Table S5. Out of the 2 studies reporting data on mean condom use, one reported a lower mean number of unprotected sex acts in the intervention group at follow up compared to the control group at follow-up, and this was a statistically significant difference [8]. The other study, which conducted 2 follow-up assessments, reported a higher number of unprotected sex acts with commercial partners among the intervention group than among the control group at the first follow-up, but ultimately fewer unprotected sex acts with commercial partners in the intervention group than in the control group at the second follow-up [31].

### Intervention effects on HIV incidence

Data pertaining to cases of HIV infection were reported in only 4 of the included studies, although one provided only baseline HIV prevalence data. Interventions assessed in these studies were peer education, participatory learning, and integrated mental health or intimate partner violence interventions along with HIV education. Results regarding HIV prevalence and incidence are shown in Table 4. Of the 3 studies reporting incident cases at follow-up, 2 reported a lower proportion of new cases in the intervention group compared to the control group (out of the number tested) [32, 33] (one assessing an integrated intimate partner violence reduction intervention and the other a participatory learning intervention), however, one, assessing a peer education intervention, reported a higher percentage of incident cases at follow-up in the intervention group [34]. For the two studies reporting HIV infection rates per 100 person years [32, 33], both reported lower rates in the intervention group compared to the control group, although significance levels were not reported.

**Table 4:** Intervention Effects on HIV Infection Outcomes

Study (Ref, Year)	Study Country	Int type	Sample Size (N)					*P* value type ^b^	HIV Prevalence and Incidence (N Cases)							
			Total N	N int ^a^	N cont	N int fu	N cont fu		int bsl (Prevalence)	int fu (Incidence)	*p* value	cont bsl (Prevalence)	Cont fu (Incidence)	*P* value	Rate per 100 py int	Rate per100 py cont
[34] 2007	Zimbabwe	PE	9454	4792	4662	6015	5993	I	1172	123	< 0.001	999	89	NR	NR	NR
[33] 2008	South Africa	PL	2776	1409	1367	1063 fu2: 1005	1006 fu2: 994	I	82	72	NR	104	81	0.56	3.46	4.07
[44] 2013	Rwanda	IMH	120	120	NA	120	NA	P	1/49	NR	NR	NA	NA	NA	NR	NA
[32] 2015 (women)	Uganda	IPV	6702	3158	3544	3775 (men and women)	4067 (men and women)	I	343/2814	56/1925	0·0026	448/3175	71/2038	0.396	0.99	1.15
[32] 2015 (men)	Uganda	IPV	4746	2179	2567			I	184/2789	27/1326	0·0288	253/2896	48/1435	0.045	0.70	1.13

*Cont* Control, *Bsl* baseline, *Fu* Follow-up, *Int* Intervention, *NR* Not reported, *PL* Participatory Learning, *PE* Peer education, *IPV* integrated intimate partner violence intervention, *IMH* integrated mental health intervention

^a^ When reported separately, total participants are shown under “sample size” and number tested is provided as the denominator for the outcome

^b^ *P* value type I = Int. vs. control, P = Pre- vs. Post-test. Where two *p* values are available, Int. vs. cont *p* values: 1st p value = Int, 2nd = Control. Pre-Post *p* values: 1st p value = pre-test, 2nd = post-test

### Influence of improvements in HIV-related knowledge on condom use

Although it is difficult to determine the precise role of increased HIV-related knowledge on subsequent sexual risk behaviour, as not all studies included in this review reported on all relevant outcomes, several observations of interest can be made in this regard. First of all, considering the possible influence of knowledge of sexual contact as a route of HIV transmission on subsequent sexual practices, of the three studies [24, 25, 30] that reported significant effects of their interventions on increased knowledge of the sexual transmission of HIV in the intervention group (Table 3), two also reported data on condom use. One reported a higher proportion of respondents always using a condom in the intervention group at follow-up compared to the control group (although no *p*-value was provided) [30], and the third [24] reported a significant increase in “any condom use” in the intervention group at follow-up compared to at baseline (*p* < 0.001) (Additional file 1: Table S5). Both of these studies implemented peer education-based interventions.

Furthermore, of the six studies reporting significant increases in knowledge of condom use as a method of HIV prevention among the intervention group [24–29], two also provided data regarding actual condom use. One of these studies has already been mentioned above, reporting a significant increase in “any condom use” in the intervention group at follow-up compared to at baseline (*p* < 0.001) [24] (Additional file 1: Table S5). The second study however reported a lower proportion of condom use in the intervention group compared to the control at follow-up (although this difference was non-significant) (Additional file 1: Table S5) [28].

### Meta-analyses

#### Knowledge of risk reduction through condom use

**Fig. 2:**
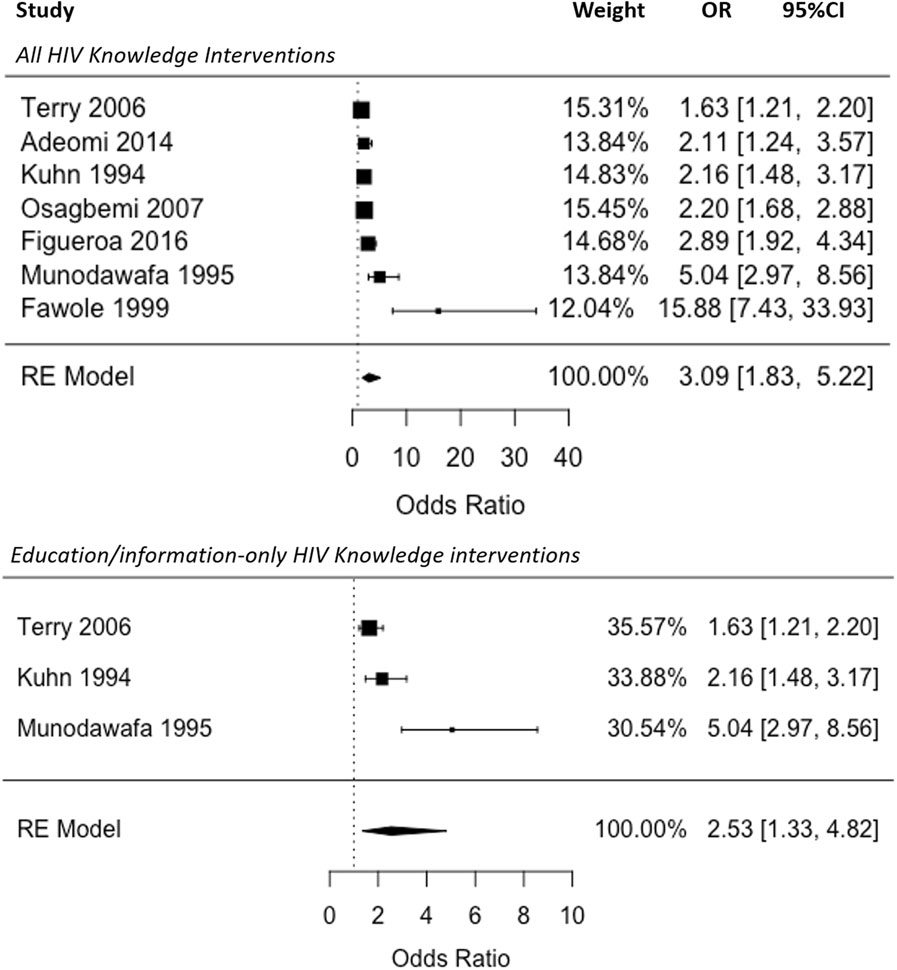
Meta-analysis: Pooled Effect of HIV Knowledge Interventions on Knowledge of HIV Risk Reduction Through Condom Use (Odds Ratios of Correct Knowledge of Risk Reduction Through Condom Use in the Control vs. Intervention Group at Follow-up)

Seven of the included studies provided post-intervention data on knowledge of risk reduction through condom use in the intervention and control groups (respondents knowing that condom use is a method of HIV risk reduction) (See Fig. 2). The intervention group had higher odds of knowing that the risk of HIV can be reduced through the use of condoms during sex than the control group in all of these studies (ORs ranging from 1.63 (95%CI: 1.21–2.20) to 15.88 (95%CI: 7.43–33.93).

The pooled OR for knowledge of condom use as a risk reduction measure across all 7 studies was 3.09, and the difference in odds of correct knowledge was significant (95%CI: 1.83–5.22, *p* < 0.0001). Separating this by intervention type, standard HIV education interventions alone (investigated in 3 studies) were also found to significantly increase the odds of correct knowledge among the intervention group (OR: 2.53, 95%CI: 1.33–4.82, *p* = 0.005), although this OR was slightly lower than the pooled OR across all 7 studies. Heterogeneity was high across the 7 studies (I^2^ = 91.53)., although slightly lower when only the three standard informational HIV knowledge interventions were considered (I^2^ = 87.59).

#### Knowledge of transmission routes

**Fig. 3:**
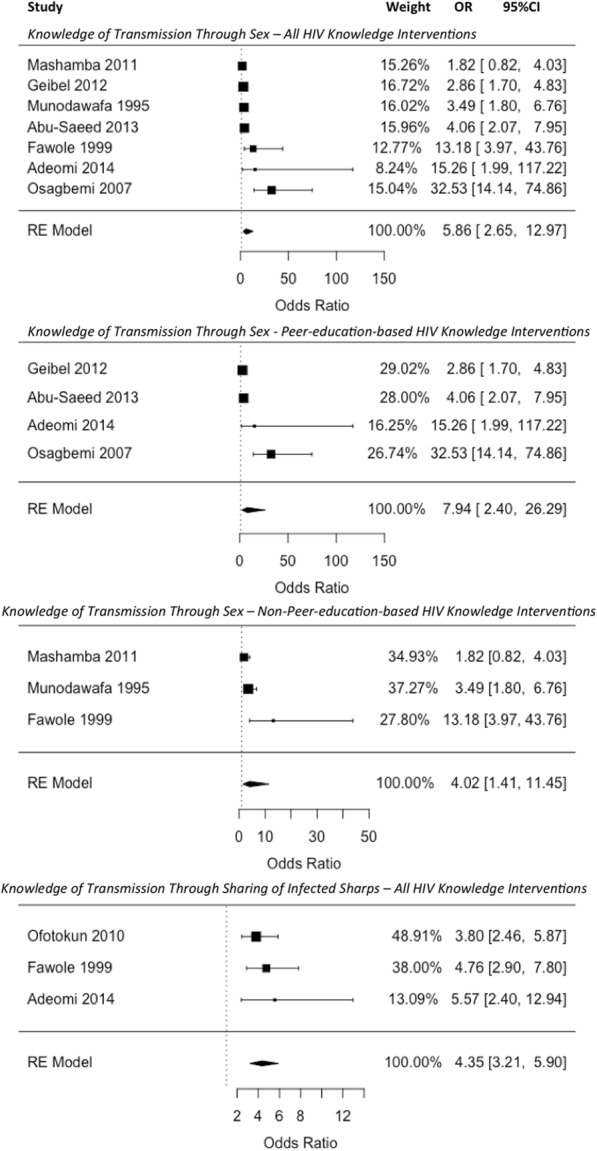
Meta-analysis: Pooled Effect of HIV Knowledge Interventions on Knowledge of HIV Transmission Routes (Odds Ratios of Correct Knowledge in the Control vs. Intervention Group at Follow-up)

As shown in Fig. 3, 7 studies provided sufficient data for inclusion in the meta-analysis of intervention effect on knowledge of HIV transmission through sex. All of these studies reported higher odds of knowledge of sexual transmission in the intervention group compared to the control group, with the intervention group being from almost twice as likely to more than 32 times as likely as the control group to know that HIV can be transmitted through sex (ORs ranging from 1.82 (95%CI: 0.82–4.03) to 32.53 (95%CI: 14.14–74.86). The pooled OR for this outcome was 5.86 (95%CI: 2.65–12.97), and these higher odds of correct knowledge among the intervention group compared to the control were significant (*p* < 0.001). When non-peer (*n* = 3) and peer-led interventions (*n* = 4) were pooled separately, both effect estimates remained significant, with higher odds of correct knowledge in the intervention group than in the control (non-peer education interventions: OR: 4.02, 95%CI: 1.41–11.45, *p* = 0.009; peer education interventions: OR: 7.94, 95%CI: 2.40–26.29, *p* < 0.001).

Regarding knowledge of transmission through sharing of infected sharps, as is common practice when the effect measure is ORs, the one study reporting a zero-event [40] was not included in the model [45]. Although the inclusion of zero-event studies has shown to provide a more conservative effect estimate in meta-analyses [45], the pooled effect estimate in this case was the same when the study was included as when it was dropped from the analysis (OR = 4.35, 95%CI = 3.21–5.89 vs. OR = 4.35, 95%CI = 3.21–5.90, respectively). The observed higher odds of correct knowledge in the intervention group for this outcome were significant (*p* < 0.001).

#### Condom use

Categorical data for condom use was not meta-analysed due to the heterogeneity in definitions of condom use, use during specific types of sexual activity or with specific types of partners, and time intervals of use, as well as due to the lack of uncertainty in the comparability across studies of measures such as “consistent condom use”. A meta-analysis of continuous data on condom use was also not carried out, as only 2 studies provided data on mean number of unprotected sex acts.

#### HIV incidence

**Fig. 4:**
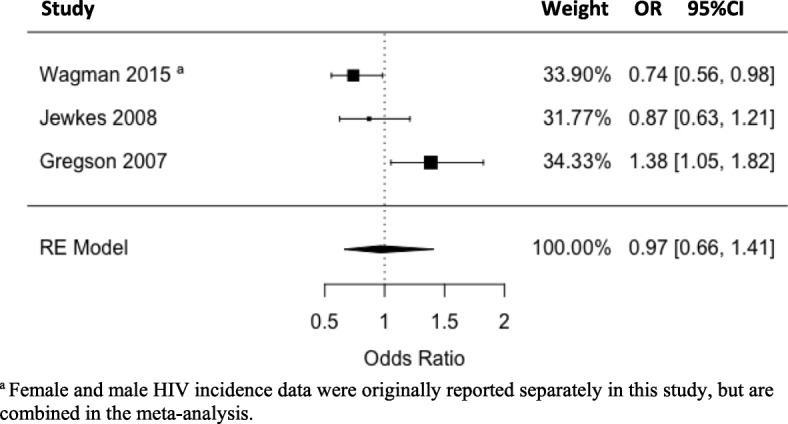
Meta-analysis: Pooled Effect of HIV Knowledge Interventions on HIV Incidence (Odds Ratio of HIV infection in Control vs. Intervention Group at Follow-up)

Figure 4 displays a meta-analysis of the 3 studies that provided post-intervention data for HIV incidence. Two studies reported lower odds of HIV infection in the intervention group compared to the control, whilst one reported higher odds in the intervention group. The pooled OR was 0.97 (95%CI: 0.66–1.41), indicating slightly lower odds of HIV infection among individuals receiving an HIV education intervention compared to those not receiving one, however, this difference was not significant (*p* = 0.854), and it should be noted that the confidence interval crosses OR = 1, indicating uncertainty regarding the true effect direction.

### Quality assessment of included studies

**Fig. 5:**
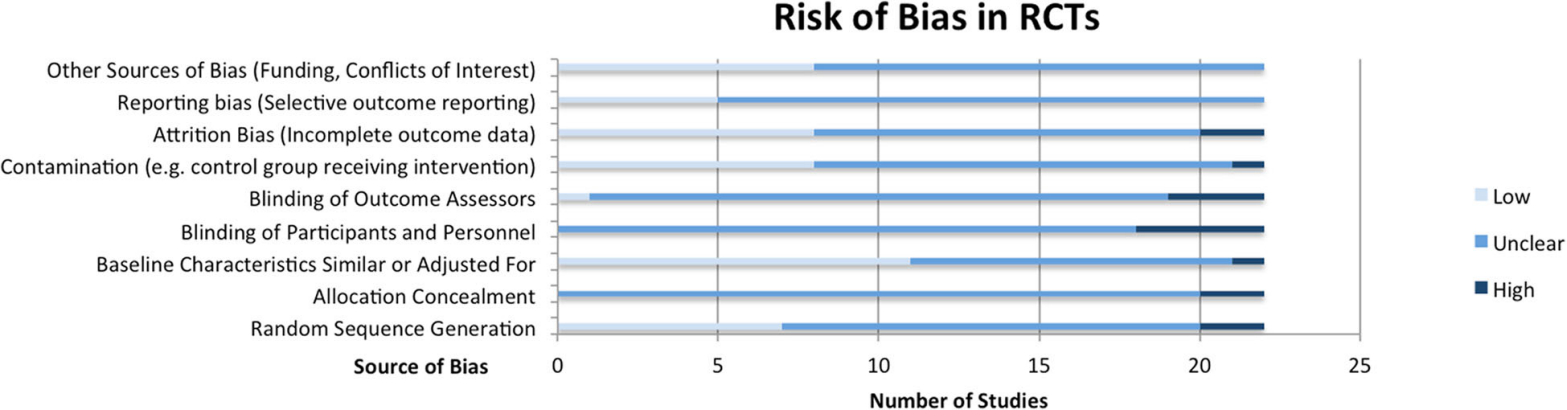
Risk of Bias Assessment for Randomized Controlled Trials

**Fig. 6:**
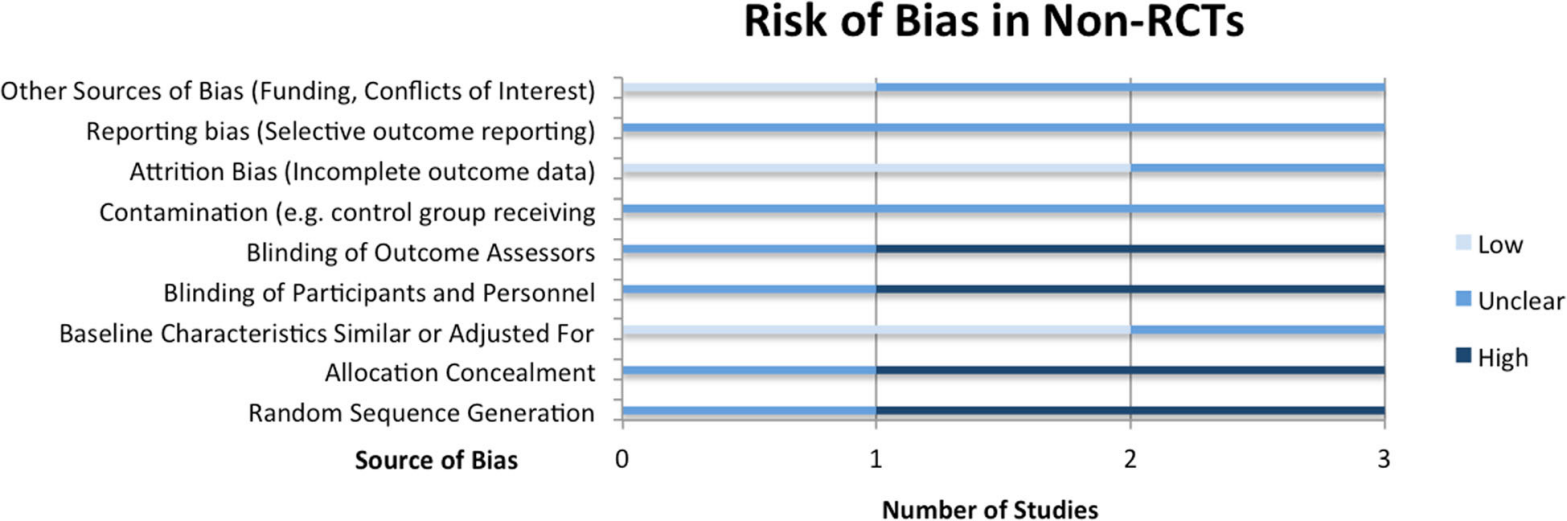
Risk of Bias Assessment for Non-randomized Controlled Trials

The methodological quality assessment of RCTs according to Cochrane Risk of Bias criteria [21] is summarized in Fig. 5. Quality assessment outcomes for non-RCTs, based on the same criteria, are shown in Fig. 6. The Cochrane Risk of Bias Tool includes criteria such as random sequence generation, measures taken to initially conceal group assignment, blinding of participants or data analysts, and measures to reduce contamination between study groups. Studies are then given a risk ranking of “low”, “unclear” or “high” risk. See Additional file 1: Table S6 for separate risk levels in each criterion for each study.

In general, RCTs were found to be of acceptable methodological quality, although a useful assessment of all criteria was not possible due to lack of detailed reporting, leading to many criteria being ranked as “unclear” in most studies. This lack of reporting was particularly true for criteria such as random sequence generation and allocation concealment, as although studies stated that group assignment was random, most did not specify how assignment was randomized (e.g. was a random number sequence generated?), and none specified how initial allocation was concealed. Moreover, most studies did not clearly mention whether participants or intervention facilitators were blinded, although given the nature of the interventions, it is reasonable to assume that blinding was not possible. A greater concern however was that it was also often not stated whether data analysts were blinded to group assignment, which would have technically been possible and should have been specifically reported. Furthermore, as most studies did not have prior registered study protocols, it was difficult to assess whether outcomes had been pre-specified and whether they had been fully reported as planned in the protocol, therefore limiting the assessment of selection bias, with only five studies being classified as “low risk” for this criterion. On the other hand, a criterion that was generally well addressed by studies was ensuring that baseline characteristics were similar between study groups, with 11 studies providing a baseline comparison and either reporting insignificant *p* values (< 0.05), or adjusting for significant differences in their subsequent analyses.

Apart from the expected higher risk scores on randomization, non-RCTs showed similar shortcomings with respect to reporting, with most not providing enough information to assess methodological quality regarding selective outcome reporting, or measures to reduce contamination.

The quality of pre-post studies was assessed using the Quality Assessment Tool for Before-After Studies with No Control Group [22] with results shown in Table 5. This assessment tool ascribes a total risk score to each study based on 11 criteria, including the recruitment of a representative sample, the justification of sample size, and appropriate reporting of attrition bias. Total risk scores are then categorized into overall rankings of low (total score 76–100%), moderate (26–75%) or high risk (0–25%). All included pre-post studies scored within the moderate risk category, with scores ranging from 45.45 to 72.73% (5/11 to 8/11). Main factors contributing to lower study quality (see Additional file 1: Table S7) were the absence of multiple before and after measures (no studies had multiple pre and post-intervention data collection points), and the inadequate reporting of attrition (loss-to-follow-up) or a lack of statistical comparison of those who completed the study to those who did not, although other criteria were well addressed across studies, such as the clear stating of study objectives and intervention activities.

**Table 5:** Risk of Bias Assessment for Pre-Post or uncontrolled studies

Ref	First Author	Year	Total Risk Score^a^ (out of 11 criteria^b^)	% score	Final Risk Level (0–25% = high risk, 26–75% = moderate risk, 76–100% = low risk)
[35]	Ajuwon	2013	6	54.55	Moderate
[39]	Klinger	2016	8	72.73	Moderate
[42]	Leonard	2000	8	72.73	Moderate
[13]	Manafa	2006	6	54.55	Moderate
[46]	Miller	2008	7	63.64	Moderate
[37]	Ndebele	2012	7	63.64	Moderate
[36]	Srinivas	2013	5	45.45	Moderate
[44]	Talbot	2013	7	63.64	Moderate
[47]	Visser	2005	5	45.45	Moderate
[48]	Temmerman	1990	6	54.55	Moderate
[41]	Geibel	2012	7	63.64	Moderate

^a^ See Additional file 1: Table S7 for specific scores on each of the 11 criteria

^b^ One of the original tool’s criteria; “in the case of group-level interventions, adjustments made for use of individual data to determine group level effects”, was not relevant to any of the included studies, and, as recommended by the tool developers, was thus not included in the assessment

Overall therefore, the concerns revealed in this risk assessment regarding the quality of the included studies are more concerns relating to the lack of sufficient detail in reporting of relevant methodological components rather than any explicitly identified methodological flaws.

## Discussion

The included studies spanned countries across Sub-Saharan Africa, [49] and assessed a wide variety of HIV-related knowledge intervention types, ranging from peer-education to video-based interventions. [12, 16, 50–57]

Regarding improving knowledge of transmission routes, peer-based educational interventions seem to be particularly effective, with all three studies that demonstrated significantly higher knowledge of sexual transmission of HIV among the intervention group having administered peer-education interventions [24, 25, 30]. Similarly, intervention types that were associated with significant improvements in knowledge of condom use as a measure of HIV risk reduction as well as increased actual condom use included peer-education, community-level education, video-based educational interventions, and standard HIV educational interventions (e.g. non-peer-led information sessions) [24–29]. Meta-analyses for knowledge outcomes showed significantly higher odds of correct knowledge among the intervention group of both, transmission through sharps (OR = 4.35, 95%CI = 3.21–5.90, *p* < 0.001), as well as through sexual intercourse (OR: 5.86, 95%CI: 2.65–12.97, *p* < 0.001). In addition, intervention participants had significantly higher odds of knowledge of condom use as a means of HIV risk reduction, and, among studies reporting actual condom use, fewer unprotected sex acts were found to occur post-intervention in the intervention groups compared to the control groups.

With regards to HIV incidence, although the meta-analysis indicated only slightly lower pooled odds of HIV infection among the intervention groups (OR: 0.97, 95%CI: 0.66–1.41, *p* = 0.854), it is interesting to note that among the two studies that reported lower HIV incidence in the intervention group, one administered an intervention integrating HIV education with intimate partner violence reduction. This suggests that addressing intimate partner violence along with HIV-related knowledge may be important for reducing sexual risk behaviour and subsequent transmission.

Although the included studies generally had low to moderate risk of bias scores, an accurate evaluation of study quality was hindered by inadequate reporting of relevant methodological elements, particularly with respect to randomization and group assignment procedures in the case of RCTs, and measures taken to reduce contamination across intervention groups.

Further limitations of this study include the reliability of the meta-analysis. Given that the included studies were published over a large period of time (1990–2016), during which both scientific understanding of and societal attitudes towards HIV changed dramatically, the pooling of results from studies across this time period is problematic, and this limitation should be taken into account when drawing conclusions regarding the effectiveness of certain interventions from the current meta-analyses. However, given that the review encompassed multiple Sub-Saharan African countries, and developments in societal attitudes towards HIV, as well as dissemination of scientific knowledge and advancements with regard to the disease reached countries - and even specific sub-populations within countries - at different times, defining a cut-off time-point for separate meta-analyses by different time periods would not be feasible. On a related note, the exclusion of qualitative studies from this review prevents the detailed consideration of how sociocultural and temporal contexts influence accessibility and uptake of HIV education interventions and their subsequent translation into preventive behaviours.

Moreover, although studies were quite homogenous and precise in their measurement methods for the meta-analysed outcomes (such as HIV incidence), considerable heterogeneity was present regarding the interventions evaluated. Although all included studies assessed HIV-related knowledge interventions, the format and mode of delivery of these educational interventions varied (e.g. peer education, video-based, or drama-based educational interventions), leading to fairly high heterogeneity in the studies (as indicated by the fact that I^2^ values were above 75% in most of the random-effects models). This therefore implies that the resulting pooled effect estimates should be interpreted with caution, and further studies reporting on the different intervention types and providing sufficient data for the outcomes of interest are required so that a sufficient number of studies will be available for separate meta-analyses for each precise format or delivery mode of HIV educational interventions.

In addition, a secondary data synthesis such as the current review has very limited capacity to accurately establish a correlation between improved HIV-related knowledge and lower HIV incidence. Therefore, further primary studies are needed that evaluate HIV-related knowledge interventions and also provide actual HIV incidence data post-intervention, as only three studies did so in the current review, limiting the conclusions that can be drawn regarding the relationship between improvements in HIV-related knowledge and ultimate HIV transmission risk. Future primary studies on HIV education interventions should collect longitudinal, individual-level data, in order to capture long-term changes in HIV-related knowledge, subsequent risk reduction behaviours, and ultimately, HIV infection, thereby allowing more accurate conclusions to be drawn regarding the relationship between HIV education and HIV incidence than the limited conclusions that can be drawn from a review on the topic.

Lastly, it must be highlighted that the reviewed interventions may have different efficacy levels in the different Sub-Saharan African countries, and their efficacy may also differ within countries by specific socioeconomic contexts or rural or urban settings. Therefore, mapping studies should be conducted prior to the implementation of any HIV-related knowledge interventions, in order to identify local needs and the most effective form of intervention dissemination in specific areas, taking into account the particular barriers to uptake each specific setting may face.

## Conclusion

In summary therefore, peer education appears to be effective in informing individuals about HIV transmission routes and risk reduction measures. Regarding actual HIV transmission, although further studies are required on the effect of improved HIV-related knowledge on ultimate transmission risk, the current review indicates that it may be of interest to incorporate not only HIV-related knowledge regarding transmission routes and risk reduction measures into HIV interventions, but rather to also include components addressing underlying sources of HIV risk other than lack of knowledge, such as issues of female disempowerment and intimate partner violence.

## Additional file

Additional file 1: **Table S1.** Search Strategy. **Table S2.** Summary of Characteristics of Included Studies. **Table S3.** Types and Components of Interventions Implemented in the Included Studies. **Table S4.** Intervention Effects on Mean Condom Use. **Table S5.** Intervention Effects on Proportion of Participants Using Condoms. **Table S6.** Risk scores across criteria for each RCT and N-RCT study (assessed via the Cochrane Risk of Bias Tool). **Table S7.** Component criteria of total risk scores for pre-post and other uncontrolled studies (assessed via the Quality Assessment Tool for Before-After (Pre-Post) Studies With No Control). (DOCX 286 kb)

## Abbreviations

AIDS: Acquired Immune Deficiency Syndrome
HIV: Human Immunodeficiency Virus
OR: Odds Ratio
RCT: Randomized Controlled Trial
SD: Standard Deviation
