# Supplementary material for: The effect of HIV educational interventions on HIV-related knowledge, condom use, and HIV incidence in sub-Saharan Africa: a systematic review and meta-analysis
Source: BMC Public Health. 2018 Nov 13;18:1254. doi: 10.1186/s12889-018-6178-y (PMC6234686; doi:10.1186/s12889-018-6178-y)
Supplement: Supplementary file 1 — Table S1. Search Strategy. Table S2. Summary of Characteristics of Included Studies. Table S3. Types and Components of Interventions Implemented in the Included Studies. Table S4. Intervention Effects on Mean Condom Use. Table S5. Intervention Effects on Proportion of Participants Using Condoms. Table S6. Risk scores across criteria for each RCT and N-RCT study (assessed via the Cochrane Risk of Bias Tool). Table S7. Component criteria of total risk scores for pre-post and other uncontrolled studies (assessed via the Quality Assessment Tool for Before-After (Pre-Post) Studies With No Control). (DOCX 286 kb) [file 12889_2018_6178_MOESM1_ESM.docx]

**Additional file 1**

**Table S1.** Search Strategy

Database: Embase Search Date: 17^th^ Nov 2017

| Search Step | Search Terms | Records Retrieved |
| --- | --- | --- |
| 1 | knowledge/ | 30872 |
| 2 | Awareness/ | 54186 |
| 3 | program development/ or education program/ or program evaluation/ | 74638 |
| 4 | intervention study/ | 35001 |
| 5 | ((Knowledge or awareness or understanding) adj5 (program* or course* or workshop* or intervention*)).ti,ab,kw. | 30779 |
| 6 | 1 or 2 | 82883 |
| 7 | 3 or 4 | 108901 |
| 8 | 6 and 7 | 3041 |
| 9 | 5 or 8 | 33279 |
| 10 | Human immunodeficiency virus/ | 101208 |
| 11 | acquired immune deficiency syndrome/ | 131585 |
| 12 | (human immunodeficiency virus or hiv or acquired immunodeficiency syndrome or AIDS).ti,ab,kw. | 457419 |
| 13 | 10 or 11 or 12 | 493085 |
| 14 | 9 and 13 | 1787 |
| 15 | "Africa south of the Sahara"/ | 11886 |
| 16 | african/ or african american/ | 80373 |
| 17 | (africa* adj4 (immigrant* or migrant* or origin* or ancestr* or american*)).ti,ab,kw. | 78814 |
| 18 ^a^ | (Sub?Sahara* Africa* or Angola or Benin or Botswana or Burkina Faso or Burundi or Cameroon or Cape Verde or Central African Republic or Chad or Comoros or Congo or Cote d?Ivoire or Eritrea or Ethiopia or Gabon or Gambia or Ghana or Guinea or Guinea-Bissau or Kenya or Lesotho or Liberia or Madagascar or Malawi or Mali or Mauritania or Mauritius or Mozambique or Namibia or Niger or Nigeria or Rwanda or Sao Tome or Senegal or Seychelles or Sierra Leone or Somalia or South Africa or South Sudan or Swaziland or Tanzania or Togo or Uganda or Zambia or Zimbabwe).ti,ab,kw. | 348116 |
| 19 | 15 or 16 or 17 or 18 | 458761 |
| 20 | 14 and 19 | 452 |

Database: Medline Search Date: 17^th^ Nov 2017

| Search Step | Search Terms | Records Retrieved |
| --- | --- | --- |
| 1 | Awareness/ | 18984 |
| 2 | knowledge/ | 10409 |
| 3 | Health Knowledge, Attitudes, Practice/ | 100226 |
| 4 | 1 or 2 or 3 | 126758 |
| 5 | Program Development/ | 28001 |
| 6 | Program Evaluation/ | 59258 |
| 7 | 5 or 6 | 78777 |
| 8 | ((Knowledge or awareness or understanding) adj5 (program* or course* or workshop* or intervention*)).ti,ab,kw. | 24313 |
| 9 | 4 and 7 | 6234 |
| 10 | 8 or 9 | 29545 |
| 11 | hiv infections/ or acquired immunodeficiency syndrome/ or hiv seropositivity/ | 259901 |
| 12 | (human immunodeficiency virus or hiv or acquired immunodeficiency syndrome or AIDS).ti,ab,kw. | 390398 |
| 13 | 11 or 12 | 421482 |
| 14 | 10 and 13 | 2099 |
| 15 | exp "africa south of the sahara"/ | 193104 |
| 16 ^a^ | (Sub?Sahara* Africa* or Angola or Benin or Botswana or Burkina Faso or Burundi or Cameroon or Cape Verde or Central African Republic or Chad or Comoros or Congo or Cote d?Ivoire or Eritrea or Ethiopia or Gabon or Gambia or Ghana or Guinea or Guinea-Bissau or Kenya or Lesotho or Liberia or Madagascar or Malawi or Mali or Mauritania or Mauritius or Mozambique or Namibia or Niger or Nigeria or Rwanda or Sao Tome or Senegal or Seychelles or Sierra Leone or Somalia or South Africa or South Sudan or Swaziland or Tanzania or Togo or Uganda or Zambia or Zimbabwe).ti,ab,kw. | 290867 |
| 17 | exp African Continental Ancestry Group/ | 84666 |
| 18 | (africa* adj4 (immigrant* or migrant* or origin* or ancestr* or american*)).ti,ab,kw. | 58033 |
| 19 | 15 or 16 or 17 or 18 | 464591 |
| 20 | 14 and 19 | 644 |

^a^ This is based on a current list of Sub-Saharan African countries, as classified by the United Nations Development Programme (49)

**Table S2.** Summary of Characteristics of Included Studies

| Ref | First Author | Year | Study Country | Study Design | Target Population | Study Setting | Total N | Age range | Type of HIV Knowledge intervention and method of dissemination | | Intervention Description | L&F | Prov. | Ctrl | FU  1 | FU  2 |
| --- | --- | --- | --- | --- | --- | --- | --- | --- | --- | --- | --- | --- | --- | --- | --- | --- |
| (30) | Abu-Saeed | 2013 | Nigeria | RCT | Adolescents &Young Adults | School | 160 | 15-24 | Peer education | G | Peer education intervention on various topics related to HIV/AIDS | 4d | PE | NR | 8w |  |
| (25) | Adeomi | 2014 | Nigeria | RCT | Adolescents&Young Adults | School | 400 | 10-19 | Peer education | G | Peer education intervention, with peer educators trained through lectures, motivational talks, and demonstrations using audiovisuals, posters, role plays, and practical demonstrations to address the reproductive health questions of students | 2w | PE | NR | 12w |  |
| (35) | Ajuwon | 2013 | Nigeria | Pre-Post | Adults | Comm | 60 | 20-69 | Community education | G | An educational intervention covering knowledge about HIV/AIDS, routes of transmission, prevention strategies, and attitude toward persons living with HIV, using posters, pamphlets, and drama presentations | 1h, one-time | LIB | No cont. (a) | NR |  |
| (31) | Bing | 2008 | Angola | RCT | Military personnel | Military bases | 568 | 18-51 | Motivational, behavioural | G | Military-focused education, motivation and behavioural HIV prevention intervention using illustrations(with monthly booster sessions) | 4.5d | FAC | Malaria intervention and shortened HIV intervention | 3m | 6m |
| (38) | Fawole | 1999 | Nigeria | Q-rct | School children | School | 450 | NR | Video / role play | G | Comprehensive reproductive health and HIV prevention education using films, role-plays, lectures | 6 w, 1 session /w for 2-6 hr | PHY | NR | 6m |  |
| (29) | Figueroa | 2016 | Mozambique | Post-only | Adults | Comm | 915 | NR | Community dialogue and video | G | A program to address underlying social determinants, especially inequitable gender norms, as a fundamental step in reducing HIV risk behaviors, increasing self-efficacy to talk about and address HIV, and decreasing HIV stigma through community discussions | 10w, 1 session /w | FAC | No cont. | NR |  |
| (50) | Futterman | 2010 | South Africa | N-rct | Women | Obstetric unit and health centre | 71 | 16-42 | Peer mentoring, Behavioural, PMTCT | G | An intervention to educate HIV+ mothers about PMTCT and to provide support through mentor mothers | NR | PE | Standard prenatal support | 6m |  |
| (41) | Geibel | 2012 | Kenya | Pre-Post | Male sex workers | Comm | 442 | 21-27 | Peer counseling | I | A drop-in centre-based HIV counseling, referral and condom distribution program | NA | PE | None | NA | NA |
| (34) | Gregson | 2007 | Zimbabwe | RCT | General Population | Comm | 9454 | NR | Peer education, income generation and clinic-based treatment and counselling | I | A program of condom distribution, STI management, HIV/AIDS educational activities and safe sex promotion | NR | PE | Standard services (including STI management) | NR |  |
| (51) | Harvey | 2000 | South Africa | RCT | Adolescents&Young Adults | School | 1080 | 13-29 | Drama-based | G | A drama-in-education HIV prevention intervention | NR | TE, AC, N | Written information about HIV/AIDS | 6m |  |
| (14) | Jemmott | 2015 | South Africa | Cluster-RCT | Adolescents&Young Adults | School | 1057 | NR | Role-play, group discussions based | G | A school based HIV prevention intervention using role playing, discussions and group exercises | 1 w, 6 2-hr sessions | NR | GH | 3m | 54m |
| (33) | Jewkes | 2008 | South Africa | Cluster-RCT | Adolescents&Young Adults | Comm | 2776 | 15-26 | Participatory leaning | G | A HIV prevention intervention to improve sexual health by using participatory learning approaches to build knowledge, risk awareness, and communication skills | 50hrs | PS | Standard HIV prevention | 12m | 24m |
| (16) | Kaponda | 2011 | Malawi | Q-rct | Adults | Comm | 1152 | NR | Peer education | G | A peer group intervention for HIV prevention aiming to foster skill building and social support among the group. Content was shaped by collaboration with local health centres | 6 2hr sessions | PE | DI | 6m | 18m |
| (39) | Klinger | 2016 | Madagascar | Pre-Post | Adolescents&Young Adults | School | 155 | 15-19 | Educational | G | A complimentary teaching methods intervention for HIV and STI prevention and reproductive health | 6 sessions, 1.5hrs each | NR | No cont. | Imm |  |
| (26) | Kuhn | 1994 | South Africa | Q-rct | Adolescents&Young Adults | School | 567 | 12-30 | Educational, active comm. participation | G | An educational HIV program designed and implemented through community participation | 2 w | TE | None | Imm |  |
| (42) | Leonard | 2000 | Senegal | Pre-Post | Truckers | Transportation parks | 260 | 13-70 | Peer education | G | A peer education program on condom use, HIV transmission and prevention by and for transport workers | NR | PE | No cont. | 3m |  |
| (13) | Manafa | 2006 | Nigeria | Pre-Post | Adults | Comm | 6000 | 18-70 | Mass media intervention | M | A mass educational campaign consisting of rallies, radio, tv and newspaper messages and workshops for teachers | NR | TE | No cont. | 3m |  |
| (40) | Mashamba | 2011 | South Africa | Q-rct | Faith healers | Church | 103 | NR | Educational | G | An educational intervention on HIV and other infectious diseases, targeted at faith healers (so that they can pass information on to their congregation) | 2 d | NR | Information booklet only | 2m |  |
| (52) | Michielsen | 2012 | Rwanda | N-RCT | Adolescents&Young Adults | School | 1950 | NR | Peer education and participatory learning | G, I | A peer-led intervention using group and individual counselling, drama performances, songs and other interactive methods. | 14 m | TE, PE | None | 6m | 12m |
| (46) | Miller | 2008 | Kenya | Pre-Post | Adolescents&Young Adults | College/Uni | 632 | NR | Peer education (ABC-based) | G | An ABC based peer education intervention for HIV prevention | 2 y | PE | No cont. | 24m |  |
| (27) | Munodawafa | 1995 | Zimbabwe | Q-rct | Adolescents&Young Adults | School | 285 | NR | Educational | G | A health education intervention provided by nursing students (focusing on HIV/AIDS and other health issues) | 7 w | NS | None | Imm |  |
| (53) | Muyinda | 2003 | Uganda | RCT | Female Adolescents&Young Adults | Comm | 95 | NR | Senga-based | G, I | An intervention training Sengas (paternal aunts) to provide HIV and sexual health information to girls in rural Uganda | 12 m | Sengas | None | 6m | 12m |
| (37) | Ndebele | 2012 | South Africa | Pre-Post | Adolescents&Young Adults | School | 130 | 14-19 | Educational | G | An educational intervention on HIV and sexual health held at regular school orientation classes | 3 w, 3 45 min sessions /w | AU | DI | 1m |  |
| (43) | Ofotokun | 2010 | Nigeria | RCT | General Population | Comm | 1205 | NR | Audio intervention, educational | G | A Culturally-adapted and audio-technology assisted HIV/AIDS awareness and education program | 1d, one-time | CC | Seminar-based HIV education | 7w |  |
| (24) | Osagbemi | 2007 | Nigeria | Q-rct | General Population | Comm | 1029 | 12-60 | Peer education, drama | G | An intervention to address the cultural norm of spouse sharing and its role in the spread of HIV, involving drama, posters, and peer education | NR | PE | None | 13m |  |
| (54) | Pronyk | 2008 | South Africa | RCT | Female Adolescents&Young Adults | Comm | 220 | 14-35 | Microcredit and HIV education | G | Group-based microfinance program plus HIV and gender equality training | 1 y, 1 session every 2 w | FAC | NR | 2y |  |
| (55) | Ross | 2006 | Nigeria | RCT | Military personnel | Military bases | 2209 | NR | Risk situation focused (role plays, discussions) | I | An individually focused intervention emphasising risk behaviour reduction | 5 modules | HE | DI | 6m | 12m |
| (36) | Srinivas | 2013 | South Africa | Pre-Post | School children | School | 432 | NR | Computer-based, board game-based | G | An HIV intervention using computer quizzes and board games, adapted to children's ages | NR | NR | No cont. | NR |  |
| (44) | Talbot | 2013 | Rwanda | Pre-Post | Adolescents&Young Adults | NGO | 120 | 15-25 | Integrated mental health intervention | G, I | An intervention for orphans, integrating both mental health services and HIV prevention | 1 y | PS | No cont. | 12m |  |
| (48) | Temmerman | 1990 | Kenya | matched control | HIV+ Mothers | Maternity wards | 1507 | NR | Nurse counseling | I | HIV+ mothers were counselled by trained nurses regarding reproductive behaviour | one-time | N | None | NR |  |
| (28) | Terry | 2006 | Zimbabwe | quasi-experimental | Adolescents&Young Adults | College/Uni | 869 | NR | Educational | G | An NGO-run health promotion and HIV prevention program | various programs | NR | None | Ongoing |  |
| (56) | Villar-Loubet | 2013 | South Africa | RCT | Pregnant couples | Community health centres | 478 (239 couples) | 18-53 | Behavioural | C | A behavioural HIV risk reduction intervention for pregnant couples in addtion to standard prenatal care | 1 m | NR | Usual prenatal care plus health education | Imm | 4-5m |
| (47) | Visser | 2005 | South Africa | Pre-Post | School children | School | 667 | NR | Life skills based | G | The intervention involves dissemination of information, development of cognitive and behavioural skills and aims at changing social norms related to sexual behaviour among young people. | 2 y | TE | No cont. | NR |  |
| (32) | Wagman | 2015 | Uganda | Cluster-RCT | General Population | Comm | 11 448 | 15-49 | Integrated Intimate partner violence prevention | M | standard of care HIV services plus a community-level mobilisation intervention to change attitudes, social norms, and behaviours related to IPV, and a screening and brief intervention to promote safe HIV disclosure and risk reduction | 4 y | PS | only standard of care HIV services | 16m | 35m |
| (8) | Wingood | 2013 | South Africa | RCT | Women | Health clinics | 342 | 18 – 35 | Peer education | G | A culturally adapted intervention addressing HIV stigma and prevention | 3 2.5hr sessions | PE | GH | 6m |  |
| (57) | Yoder | 1996 | Zambia | Q-rct | General Population | Comm | 1600 | NR | Radio drama broadcast | M | A national radio drama about HIV prevention | 9 m | NA | low radio access group | Imm |  |

AC: Actors AU: Authors C: Couple Comm.: Community

Ctrl: Control group treatment d: day(s) DI: Delayed intervention FAC: Facilitators

FU1&2: Follow-up time 1&2 G: Group GH: General health intervention GS: Graduate students

HE: Health educators I: Individual Imm: Immediate L&F: Length and frequency of intervention

LIB: Librarians M: Mass / community campaign m: month N: Nurses

N-RCT: non-RCT NS: Nursing Students PE: Peer educators PHY: Physicians

Prov.: Providers of intervention PS: Program Staff Q-RCT: Quasi-RCT RCT: Randomized Controlled Trial

TE: Teachers TRA: Trainers

(a) no cont. = study had no control

(b) none = no treatment

**Table S3.** Types and Components of Interventions Implemented in the Included Studies

| **Study**  **(Ref, Author, Year)** | | **Intervention Type / Components** | | | | | | | | | | | | | | | | |
| --- | --- | --- | --- | --- | --- | --- | --- | --- | --- | --- | --- | --- | --- | --- | --- | --- | --- | --- |
|  |  | **Audio** | **Behaviour change** | **Community education** | **Computer-based** | **Skills-based** | **Drama** | **Educational /**  **informational** | **Microcredit** | **Integrated psychosocial**  **intervention** | **Mass media campaign** | **Motivational and**  **Behavioural** | **Peer education** | **PMTCT** | **Radio** | **Scenarios / situational**  **/ role plays** | **Video** | **Others** |
| (30) Abu-Saeed | 2013 |  |  |  |  |  |  |  |  |  |  |  |  |  |  |  |  |  |
| (25) Adeomi | 2014 |  |  |  |  |  |  |  |  |  |  |  |  |  |  |  |  |  |
| (35) Ajuwon | 2013 |  |  |  |  |  |  |  |  |  |  |  |  |  |  |  |  |  |
| (31) Bing | 2008 |  |  |  |  |  |  |  |  |  |  |  |  |  |  |  |  |  |
| (38) Fawole | 1999 |  |  |  |  |  |  |  |  |  |  |  |  |  |  |  |  |  |
| (29) Figueroa | 2016 |  |  |  |  |  |  |  |  |  |  |  |  |  |  |  |  |  |
| (50) Futterman | 2010 |  |  |  |  |  |  |  |  |  |  |  |  |  |  |  |  |  |
| (41) Geibel | 2012 |  |  |  |  |  |  |  |  |  |  |  |  |  |  |  |  |  |
| (34) Gregson | 2007 |  |  |  |  |  |  |  |  |  |  |  |  |  |  |  |  |  |
| (51) Harvey | 2000 |  |  |  |  |  |  |  |  |  |  |  |  |  |  |  |  |  |
| (14) Jemmott | 2015 |  |  |  |  |  |  |  |  |  |  |  |  |  |  |  |  |  |
| (33) Jewkes | 2008 |  |  |  |  |  |  |  |  |  |  |  |  |  |  |  |  | Participatory learning |
| (16) Kaponda | 2011 |  |  |  |  |  |  |  |  |  |  |  |  |  |  |  |  |  |
| (39) Klinger | 2016 |  |  |  |  |  |  |  |  |  |  |  |  |  |  |  |  |  |
| (26) Kuhn | 1994 |  |  |  |  |  |  |  |  |  |  |  |  |  |  |  |  |  |
| (42) Leonard | 2000 |  |  |  |  |  |  |  |  |  |  |  |  |  |  |  |  |  |
| (13) Manafa | 2006 |  |  |  |  |  |  |  |  |  |  |  |  |  |  |  |  |  |
| (40) Mashamba | 2011 |  |  |  |  |  |  |  |  |  |  |  |  |  |  |  |  |  |
| (52) Michielsen | 2012 |  |  |  |  |  |  |  |  |  |  |  |  |  |  |  |  |  |
| (46) Miller | 2008 |  |  |  |  |  |  |  |  |  |  |  |  |  |  |  |  |  |
| (27) Munodawafa | 1995 |  |  |  |  |  |  |  |  |  |  |  |  |  |  |  |  |  |
| (53) Muyinda | 2003 |  |  |  |  |  |  |  |  |  |  |  |  |  |  |  |  | “Senga”-based (a) |
| (37) Ndebele | 2012 |  |  |  |  |  |  |  |  |  |  |  |  |  |  |  |  |  |
| (43) Ofotokun | 2010 |  |  |  |  |  |  |  |  |  |  |  |  |  |  |  |  |  |
| (24) Osagbemi | 2007 |  |  |  |  |  |  |  |  |  |  |  |  |  |  |  |  |  |
| (54) Pronyk | 2008 |  |  |  |  |  |  |  |  |  |  |  |  |  |  |  |  |  |
| (55) Ross | 2006 |  |  |  |  |  |  |  |  |  |  |  |  |  |  |  |  |  |
| (36) Srinivas | 2013 |  |  |  |  |  |  |  |  |  |  |  |  |  |  |  |  |  |
| (44) Talbot | 2013 |  |  |  |  |  |  |  |  | (b) |  |  |  |  |  |  |  |  |
| (48) Temmerman | 1990 |  |  |  |  |  |  |  |  |  |  |  |  |  |  |  |  | Nurse-led counselling |
| (28) Terry | 2006 |  |  |  |  |  |  |  |  |  |  |  |  |  |  |  |  |  |
| (56) Villar-Loubet | 2013 |  |  |  |  |  |  |  |  |  |  |  |  |  |  |  |  |  |
| (47) Visser | 2005 |  |  |  |  |  |  |  |  |  |  |  |  |  |  |  |  |  |
| (32) Wagman | 2015 |  |  |  |  |  |  |  |  | (c) |  |  |  |  |  |  |  |  |
| (8) Wingood | 2013 |  |  |  |  |  |  |  |  |  |  |  |  |  |  |  |  |  |
| (57) Yoder | 1996 |  |  |  |  |  |  |  |  |  |  |  |  |  |  |  |  |  |

Blacked-out fields indicate components part of each intervention.

PMTCT = Prevention of Mother-to-Child Transmission

(a) “Senga” is the Ugandan term for a father's sister, and this intervention was based on the traditional Ugandan societal concept that a young female's paternal sister is considered her main source of information regarding marriage and sexual relationships

(b) Integrated mental health intervention

(c) Integrated intimate partner violence intervention

**Table S4.** Intervention Effects on Mean Condom Use

| Study (Ref, Year) | Study Country | Int type | Sample Size (N) | | | | | P value type ^a^ | Measure / definition of condom use | Time Frame | Condom Use (Mean, SD) | | | | | | | | | |
| --- | --- | --- | --- | --- | --- | --- | --- | --- | --- | --- | --- | --- | --- | --- | --- | --- | --- | --- | --- | --- |
|  |  |  | Total | int | cont | int fu | cont fu |  |  |  | Int bsl | SD | int fu | SD | p value | Cont bsl | SD | Cont fu | SD | p value |
| (31) 2008 | Angola | Motiv / BC | 568 | 280 | 288 | NR | NR | NA | Number of unprotected vaginal sex acts with girlfriends | 3m | 2.1 | 4.6 | 1.2 fu2: 1.6 | 2.5 fu2: 4.0 |  | 2.2 | 4.8 | 2.0  fu2: 1.8 | 4.5  fu2:  4.7 |  |
| (31) 2008 | Angola | Motiv / BC | 568 | 280 | 288 | NR | NR | NA | Number of unprotected vaginal sex acts with occasional partners | 3m | 0.5 | 1.7 | 0.2 fu2: 0.1 | 1.2 fu2: 0.9 |  | 0.7 | 2.6 | 0.4  fu2:  0.2 | 2.4  fu2:  1.0 |  |
| (31) 2008 | Angola | Motiv / BC | 568 | 280 | 288 | NR | NR | NA | Number of unprotected vaginal sex acts with commercial partners | 3m | 0.2 | 1.2 | 0.1 fu2: 0.04 | 0.6 fu2: 0.4 |  | 0.2 | 2 | 0.04  fu2:  0.2 | 0.4  fu2:  1.8 |  |
| (8)  2013 | South Africa | PE | 342 | 175 | 167 | 121 | 120 | I | Number of unprotected vaginal sex acts | 3m | 6.92 | 10.03 | 4.01 | 0.9 | 0.73 | 6.52 | 11.08 | 6.57 | 0.94 | 0.05 (fu int vs cont) |

BC = Behaviour change Bsl = baseline Cont = Control Fu = Follow-up Int = Intervention

Motiv = Motivational NR = Not reported PE = Peer education

^a^ P value type I= Int. vs. control, P = Pre- vs. Post-test. Where two p values are available, Int. vs. cont p values: 1^st^ p value =Int, 2^nd^=Control. Pre-Post p values: 1^st^ p value=pre-test, 2^nd^ = post-test.

**Table S5.** Intervention Effects on Proportion of Participants Using Condoms

| Study (Ref, Year) | Study Country | Int type | Sample Size (N) | | | | | P value type ^c^ | Measure / definition of condom use | Proportion of Condom use (N of respondents or N sex acts, as specified) | | | | | | | |
| --- | --- | --- | --- | --- | --- | --- | --- | --- | --- | --- | --- | --- | --- | --- | --- | --- | --- |
|  |  |  | Total | int bsl ^a^ | cont bsl | int fu | cont fu |  |  | int bsl | int fu | SD | P value | cont bsl | cont fu | SD | P value |
| (56) 2013 | South Africa | BC | 159 | ^b^ |  |  |  | P | Consistent condom use in the past 7 days | 27% | 60% fu2: 43% |  | 0.9 | 21% | 20% fu2: 33% |  | 0.95 |
| (40) 2011 | South Africa | Ed | 103 | 58 | 45 | 58 | 45 | NA | Any condom use (unspecified period) | 36 | 40 |  | NR | 40 | 42 |  | NR |
| (46) 2008 | Kenya | Ed | 632 | 632 |  | 746 |  | P | Ever used a condom (of those who have ever had sex) | 77% | 86% |  | <0.001 |  |  |  |  |
| (46) 2008 | Kenya | Ed | 632 | 632 |  | 746 |  | P | Used a condom every time had sex in previous month | 40% | 45% |  | <0.05 |  |  |  |  |
| (28) 2006 | Zimbabwe | Ed | 869 | NR | NR | 251 | 618 | I | Used a condom at last sex (of those who have ever had sex, n=191 int and n=512 cont | NR | 56 |  | ns | NR | 182 |  |  |
| (54) 2008 | South Africa | Ed / micro | 220 | 60 | 59 | 51 | 45 | NA | Unprotected sex with a non-spousal partner in the past 12 months | 40 | 28 |  | NR | 47 | 35 |  | NR |
| (44) 2013 | Rwanda | IMH | 120 | 120 |  | 120 |  | P | Use of condoms when engaged in high-risk sexual behaviour | 65 | 94 |  | NR |  |  |  |  |
| (32) 2015 | Uganda | IPV | 11448.00 | 5337 | 6111 | 2282 fu2: 931 | 2362 fu2: 1170 | I | Condom use in past year (female data) | NR | 216 fu2: 157 |  |  | NR | 201 fu2: 192 |  |  |
| (32) 2015 | Uganda | IPV | 11448.00 | 5337 | 6111 | 1408 fu2: 601 | 1613 fu2: 806 | I | Condom use in past year (male data) |  | 216 fu2: 153 |  |  |  | 217 fu2: 188 |  |  |
| (13) 2006 | Nigeria | MM | 6000 | 6000 |  | NR |  | NA | Use condoms during sex | 38.4% | 46.9% |  |  |  |  |  |  |
| (30) 2013 | Nigeria | PE | 160 | 80 | 80 | 80 | 80 | I | Always use a condom (n) | 5 | 12 |  |  | 2 | 2 |  | NR |
| (41) 2012 | Kenya | PE | 442 |  |  | 145 | 297 | I | Always used a condom with clients in past 30 days |  | 90 |  |  |  | 132 |  |  |
| (41) 2012 | Kenya | PE | 205 |  |  | 80 | 125 | I | Always used a condom with non-paying clients in the past 30 days |  | 37 |  |  |  | 44 |  |  |
| (16) 2011 | Malawi | PE | 1152 | 629 | 523 | 180 fu2: 415 | 176 fu2: 413 | I | Ever used a condom in the past 2 months | NR | 36 fu2: 77 |  | <0.01 | NR | 13 fu2: 40 |  | <0.01 |
| (42) 2000 | Senegal | PE | 260 | 48 |  | 48 |  | P | Always uses a condom with regular partners | 25 | 42 |  | NR |  |  |  |  |
| (50) 2010 | South Africa | PE / BC / MTC | 71 | 40 | 31 | 40 | 31 | NA | Always use a condom or abstaining | NR | 38 |  |  | NR | 30 |  |  |
| (24) 2007 | Nigeria | PE / drama | 1029 | 591 | 438 | 588 | 430 | P | Any condom use | 18 | 48 |  | <0.001 | 14 | 17 |  | NR |
| (52) 2012 | Rwanda | PE / PL | 1950 | 186 | 124 | 135 fu2: 133 | 109 fu2: 108 | NA | Used condom at last sex (of those sexually active) | 83 | 55 fu2: 63 |  | NR | 47 | 45 fu2: 44 |  | NR |
| (55) 2006 | Nigeria | scenario | 2209 | 1222 | 987 | 825 fu2: 884 | 811 fu2: 875 | NA | vaginal or anal sx without condoms with a casual partner in the past 6 weeks | 1081 | 507 fu2: 601 |  | NR | 908 | 743 fu2: 840 |  | NR |
| (47) 2005 | South Africa | Skills | 667 | 33 |  | 43 |  | P | No condom use at last sex | 33 | 36 |  | 0.03 |  |  |  |  |
| (14) 2015 | South Africa | Self-E | 1056 | 561 | 495 | 543 fu2: 519 | 485 fu2: 455 | NA | Any unprotected vaginal intercourse in the past 3 months | 4 | 9 fu2: 132 |  | NR | 3 | 20 fu2: 126 |  | NR |
| (38) 1999 | Nigeria | Video / scenario | 450 | 233 | 217 | 223 | 210 | NA | Always use a condom |  | 11 | NR |  |  | 10 | NR |  |
| (51) 2000 | South Africa | Drama | 1080 | 487 | 576 | 298 | 380 | NA | Ever used a condom (of those who have had sex) | 165 | 129 |  | NR | 193 | 188 |  | NR |
| (48) 1990 | Kenya | NC | 1507 | 94 | 1413 | 24 | 33 | I | Any condom use | NR | 2 |  | ns | NR | 2 |  |  |
| (33) 2008 | South Africa | PL | 2776 | 1409 | 1367 | 1063 fu2: 1005 | 1006 fu2: 994 | I |  |  |  |  |  |  |  |  |  |
| (57) 1996 | Zambia | Radio | 1600 | 314 | 407 | 374 | 412 | P | Ever used a condom (male data) | 49.7% | 50.5% |  | NR | 29% | 36.4% |  | <0.05 |
| (57) 1996 | Zambia | Radio | 1600 | 278 | 530 | 267 | 548 | P | Ever used a condom (female data) | 20.8% | 27.0% |  | NR | 10.9% | 20.3% |  | <0.001 |
| (53) 2003 | Uganda | Senga ^d^ | 95 | 26 | 11 | 26 fu2: 26 | 11 fu2: 11 | NA | Consistent condom use (of sexually active sample) | 13 | 14 fu2: 19 |  | NR | 5 | 6  fu2:  6 |  | NR |

BC = Behaviour change Bsl = baseline CE = Community Education Comp = Computer-based

Cont = Control Ed = Educational / informational Fu = Follow-up

IMH = integrated mental health intervention Int = Intervention IPV = integrated intimate partner violence intervention

Micro = Microcredit loans MM = Mass Media MTC = Mother-to-Child Transmission Prevention

NC = Nurse counselling NR = Not reported PE = Peer education

PL = Participatory Learning Scenario = scenario-based /role-play based Self-E = Self-efficacy based

Skills = Skill-building based

^a^ Only N values for the sexually active subsamples relevant to condom use outcomes are reported for all treatment arm sample sizes here.

^b^ N for treatment arms unclear for this outcome, as sexually active proportion is not reported.

^c^ P value type I= Int. vs. control, P = Pre- vs. Post-test. Where two p values are available, Int. vs. cont p values: 1^st^ p value =Int, 2^nd^=Control. Pre-Post p values: 1^st^ p value=pre-test, 2^nd^ = post-test.

^d^ “Senga” is the Ugandan term for a father's sister, and this intervention was based on the traditional Ugandan societal concept that a young female's paternal sister is considered her main source of information regarding marriage and sexual relationships

**Table S6.** Risk scores across criteria for each RCT and N-RCT study (assessed via the Cochrane Risk of Bias Tool).

**Risk of Bias Level:**

| **Low** | **Unclear** | **High** |
| --- | --- | --- |

| **First Author** | **Year** | **Study Design** | **Risk of Bias Criteria** | | | | | | | | |
| --- | --- | --- | --- | --- | --- | --- | --- | --- | --- | --- | --- |
|  |  |  | **Random Sequence Generation** | **Allocation Concealment** | **Baseline Characteristics Similar (control vs intervention group)** | **Blinding of participants and personnel** | **Blinding of Outcome Assessors** | **Contamination** | **Attrition bias (incomplete outcome data)** | **Reporting bias (Selective outcome reporting)** | **Other Sources of Bias (Funding, COIs)** |
| Jemmott | 2015 | Cluster-RCT | Unclear | Unclear | Low | High | Unclear | Low | Low | Low | Unclear |
| Jewkes | 2008 | Cluster-RCT | Low | Unclear | Unclear | High | High | Low | Unclear | Low | Low |
| Wagman | 2015 | Cluster-RCT | Low | Unclear | Low | Unclear | Unclear | Unclear | High | Low | Low |
| Fawole | 1999 | Q-rct | High | Unclear | Low | Unclear | Unclear | Unclear | Unclear | Unclear | Unclear |
| Kaponda | 2011 | Q-rct | Unclear | Unclear | Low | Unclear | Unclear | Low | Low | Unclear | Low |
| Kuhn | 1994 | Q-rct | Unclear | Unclear | Unclear | Unclear | Unclear | Low | Low | Unclear | Unclear |
| Mashamba | 2011 | Q-rct | Unclear | Unclear | High | Unclear | Unclear | Unclear | Low | Unclear | Low |
| Munodawafa | 1995 | Q-rct | Unclear | Unclear | Unclear | Unclear | Unclear | Unclear | Unclear | Unclear | Unclear |
| Osagbemi | 2007 | Q-rct | Unclear | Unclear | Unclear | Unclear | Unclear | Low | Unclear | Unclear | Unclear |
| Yoder | 1996 | Q-rct | Low | Unclear | Unclear | High | High | High | Unclear | Unclear | Unclear |
| Abu-Saeed | 2013 | RCT | Low | Unclear | Unclear | Unclear | Unclear | Low | Unclear | Unclear | Unclear |
| Adeomi | 2014 | RCT | Unclear | Unclear | Low | Unclear | Unclear | Unclear | Unclear | Unclear | Low |
| Bing | 2008 | RCT | Low | Unclear | Low | Unclear | Unclear | Low | Low | Unclear | Unclear |
| Gregson | 2007 | RCT | Low | High | Low | High | High | Unclear | Low | Low | Unclear |
| Harvey | 2000 | RCT | Unclear | Unclear | Low | Unclear | Unclear | Unclear | High | Unclear | Unclear |
| Muyinda | 2003 | RCT | Unclear | Unclear | Unclear | Unclear | Unclear | Unclear | Unclear | Unclear | Unclear |
| Ofotokun | 2010 | RCT | High | High | Unclear | Unclear | Unclear | Low | Unclear | Unclear | Low |
| Pronyk | 2008 | RCT | Unclear | Unclear | Low | Unclear | Unclear | Unclear | Low | Low | Low |
| Ross | 2006 | RCT | Low | Unclear | Low | Unclear | Unclear | Unclear | Unclear | Unclear | Unclear |
| Villar-Loubet | 2013 | RCT | Unclear | Unclear | Unclear | Unclear | Unclear | Unclear | Unclear | Unclear | Unclear |
| Figueroa | 2016 | RCT | Unclear | Unclear | Low | Unclear | Unclear | Unclear | Unclear | Unclear | Unclear |
| Wingood | 2013 | RCT | Unclear | Unclear | Unclear | Unclear | Low | Unclear | Low | Unclear | Low |
| Futterman | 2010 | N-rct | Unclear | Unclear | Low | Unclear | Unclear | Unclear | Low | Unclear | unclear |
| Michielsen | 2012 | N-rct | High | High | Low | High | High | Unclear | Unclear | Unclear | low |
| Terry | 2006 | Quasi-experimental | High | High | Unclear | High | High | Unclear | Low | Unclear | unclear |

**Table S7.** Component criteria of total risk scores for pre-post and other uncontrolled studies (assessed via the Quality Assessment Tool for Before-After (Pre-Post) Studies With No Control).

| First Author | Year | Objective Clearly Stated | Eligibility criteria for participants pre-specified and clear | Participants representative of the general population of interest | Unbiased enrollment of all eligible participants | Sample size sufficient (confirmed by power calculation) | Intervention clearly described and consistently delivered to all participants | Outcomes pre-specified and consistently measured | Blinding of outcome assessors | Loss to follow-up less than 20% or accounted for in the analysis | Statistical methods appropriate to detect changes before to after | Multiple before and after measures | Total Score, out of 11 ^a^  (% score) | Final Risk Level ^b^ |
| --- | --- | --- | --- | --- | --- | --- | --- | --- | --- | --- | --- | --- | --- | --- |
| Ajuwon | 2013 | 1 | 0 | 1 | 0 | 0 | 1 | 1 | 0 | 1 | 1 | 0 | 6 (54.55) | M |
| Klinger | 2016 | 1 | 1 | 1 | 1 | 0 | 1 | 1 | 0 | 1 | 1 | 0 | 8 (72.73) | M |
| Leonard | 2000 | 1 | 1 | 1 | 1 | 0 | 1 | 1 | 0 | 1 | 1 | 0 | 8 (72.73) | M |
| Manafa | 2006 | 1 | 0 | 1 | 1 | 0 | 1 | 1 | 0 | 0 | 1 | 0 | 6 (54.55) | M |
| Miller | 2008 | 1 | 1 | 1 | 1 | 0 | 1 | 1 | 0 | 0 | 1 | 0 | 7 (63.64) | M |
| Ndebele | 2012 | 1 | 1 | 0 | 1 | 1 | 1 | 1 | 0 | 0 | 1 | 0 | 7 (63.64) | M |
| Srinivas | 2013 | 1 | 0 | 0 | 1 | 0 | 1 | 1 | 0 | 0 | 1 | 0 | 5 (45.45) | M |
| Talbot | 2013 | 1 | 1 | 1 | 1 | 0 | 1 | 1 | 0 | 0 | 1 | 0 | 7 (63.64) | M |
| Visser | 2005 | 1 | 0 | 1 | 0 | 0 | 1 | 1 | 0 | 0 | 1 | 0 | 5 (45.45) | M |
| Temmerman | 1990 | 1 | 1 | 0 | 1 | 0 | 1 | 1 | 0 | 0 | 1 | 0 | 6 (54.55) | M |
| Geibel | 2012 | 1 | 1 | 0 | 1 | 0 | 1 | 1 | 0 | 1 | 1 | 0 | 7 (63.64) | M |

^a^ One of the original tool’s criteria; “in the case of group-level interventions, adjustments made for use of individual data to determine group level effects”, was not relevant to any of the included studies, and, as recommended by the tool developers, was thus not included in the assessment.

^b^ 0-25% = high risk, 26-75% = moderate risk (M), 76-100% = low risk
